# Development of Amniotic Epithelial Stem Cells Secretome-Loaded *In Situ* Inverse Electron Demand Diels–Alder-Cross-Linked Hydrogel as a Potential Immunomodulatory Therapeutical Tool

**DOI:** 10.1021/acsami.4c16659

**Published:** 2025-01-02

**Authors:** Rubén Pareja Tello, Adrián Cerveró-Varona, Giuseppe Prencipe, Giuseppina Molinaro, Veronica Pinnarò, Arlette Alina Haidar-Montes, Alexandra Correia, Sami Hietala, Johannes Stöckl, Jouni Hirvonen, Goncalo Barreto, Valentina Russo, Barbara Barboni, Hélder A. Santos

**Affiliations:** † Drug Research Program, Division of Pharmaceutical Chemistry and Technology, University of Helsinki, 00014 Helsinki, Finland; ‡ Unit of Basic and Applied Sciences, Department of Biosciences and Agro-Food and Environmental Technologies, University of Teramo, 64100 Teramo, Italy; § Center for Pathophysiology, Infectiology and Immunology, Institute of Immunology, Medical University of Vienna, 1090 Vienna, Austria; ∥ Department of Chemistry, University of Helsinki, 00014 Helsinki, Finland; ⊥ Clinicum, Faculty of Medicine, University of Helsinki and Helsinki University Hospital, 00014 Helsinki, Finland; # Medical Ultrasonics Laboratory (MEDUSA), Department of Neuroscience and Biomedical Engineering, Aalto University, 02150 Espoo, Finland; ∇ Orton Orthopedic Hospital, Tenholantie 10, 00280 Helsinki, Finland; ○ Department of Biomaterials and Biomedical Technology, The Personalized Medicine Research Institute (PRECISION), University Medical Center Groningen, University of Groningen, Ant. Deusinglaan 1, 9713 AV Groningen, The Netherlands

**Keywords:** hydrogel, stem cells, secretome, click chemistry, regenerative medicine

## Abstract

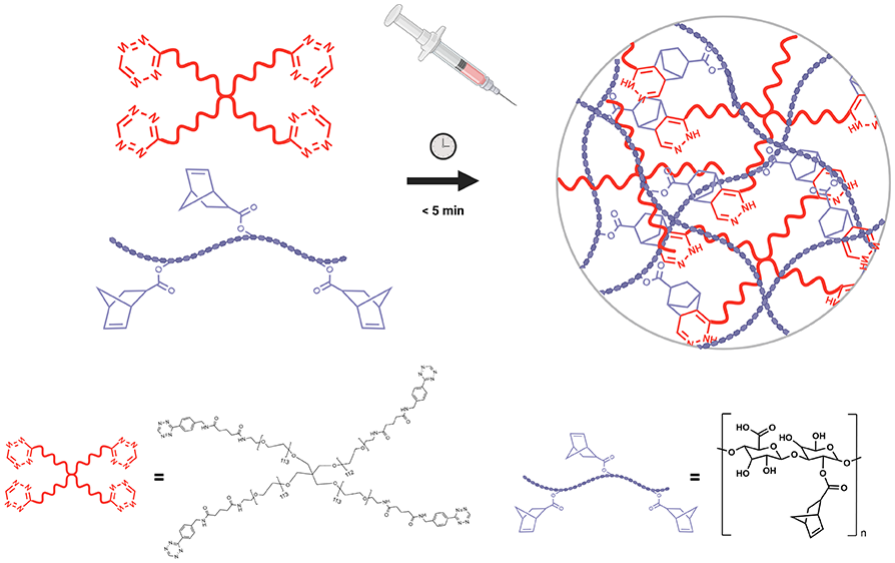

Amniotic epithelial stem cells (AEC) hold potential for tissue regeneration, especially through their conditioned medium (AEC-CM) due to their immunomodulatory and regenerative effects. Nevertheless, advanced drug delivery systems such as hydrogels are needed to enable clinical applications. Herein, an *in situ* gellable hyaluronic acid and polyethylene glycol-based iEDDA-cross-linked hydrogel was developed for the encapsulation and controlled release of AEC-CM. The developed system was formed by norbornene-modified hyaluronic acid and tetrazine-modified polyethylene glycol functionalized with heparin. The hydrogel was formed by mixing both precursor polymers, displaying fast cross-linking kinetics and showcasing a highly porous inner structure and low swelling properties. Moreover, the heparin-functionalized system allowed the sustained release of predominant growth factors from AEC-CM over 14 days. *In vitro* studies in peripheral blood mononuclear cells (PBMCs) showed an enhanced suppression efficacy and a significant shift toward the M2 macrophage phenotype in comparison with nonencapsulated AEC-CM. Therefore, this work provides a suitable alternative for the encapsulation of AEC-CM in a hydrogel formulation, highlighting its potential as an alternative immunomodulatory therapeutic tool for tissue regeneration.

## Introduction

Amniotic epithelial stem cells (AEC), originally derived from the inner layer of the placenta, are a highly promising tool for the development of novel therapeutic treatments for tissue regeneration.^1−3^ These cells exhibit significant proliferation properties and differentiation potential, remain widely accessible, and pose minimal ethical concerns.^2^ These cells are unique due to their pluripotent nature, allowing them to differentiate into various cell types, and their immunomodulatory properties, which help modulate immune responses and promote healing.^4^

The capacity of AEC to improve healing processes by direct differentiation, cell-to-cell communication, and paracrine activities is a crucial component of their effectiveness.^5^ Furthermore, by shifting inflammation toward a pro-regenerative immunological profile, AEC have demonstrated efficacy in circumstances marked by persistent inflammatory responses.^6^ Immunomodulatory molecules are specifically released to accomplish this immune-modulating function, which successfully modifies the inflammatory milieu and stimulates tissue regeneration.^7^ By utilizing the variety of growth factors, cytokines, and extracellular vesicles that these cells release, AEC-conditioned medium (CM) has become a viable cell-free method.^8^ These factors positively coordinate inflammation and healing processes with immune cells and stromal/progenitor cells, improving tissue functionality and cellular activities.^9,10^

However, a significant challenge remains in the cell-free use of AEC without a clearly defined method for their delivery. Traditional delivery methods like direct injection often result in rapid clearance from the target site, limited bioavailability, and suboptimal therapeutic outcomes.^11^ Developing new technologies and techniques is essential to address this gap and make AEC-CM therapeutics viable for clinical applications. A key consideration is the need for controlled and long-lasting release of the loaded molecules, which is vital for finely tuning and managing the inflammatory host response.^12,13^ Innovative delivery systems and methods are being explored to ensure the efficient and targeted application of AEC-CM, thereby enhancing its therapeutic potential and facilitating its translation into clinical practice.^14^

Among the available technologies, injectable hydrogels are valuable tools in tissue engineering and drug delivery, capable of delivering specific drug cargos and simultaneously enhancing the regeneration of functional tissue.^15^ These systems can be used for the controlled delivery of novel biological cargos by selecting appropriate materials that interact with the loaded cargo. The formulation’s loading efficiency and release kinetics can be modified by the internal network characteristics, surface area, electrostatic properties, and the addition of functional moieties into the hydrogel matrix.^16,17^ Among different cross-linking strategies, inverse electron demand Diels–Alder (iEDDA) click chemistry between tetrazine and norbornene is highly suitable for developing covalently cross-linked hydrogels due to its high chemoselectivity, high efficiency, and fast cross-linking kinetics.^18,19^ This approach offers significant advantages, including compatibility with biological payloads and the lack of need for additional agents, making iEDDA highly suitable for developing injectable hydrogels.^20^ Recent studies have demonstrated the feasibility of producing iEDDA-cross-linked hydrogel microspheres with high encapsulation efficiency and noncross reactivity with the loaded cargo.^21^

Numerous synthetic and natural biomaterials have been used for the development of hydrogel formulations with the aim to encapsulate highly complex biological conditioned media.^22^ Hyaluronic acid (HA), an extracellular matrix (ECM)-derived nonsulfated glycosaminoglycan, has been frequently used as common natural-derived biomaterial due to its well-known biocompatibility and biodegradability.^23^ Furthermore, polyethylene glycol (PEG) constitutes a widely utilized synthetic polymer with high biocompatibility and suitable mechanical properties.^24^ Since the combination of both materials is characterized by the formation of interconnected polymer networks with high permeability in aqueous media, the use of these systems for the encapsulation, retention, and prolonged release of hydrophilic cargos remains challenging.^25^

Several studies suggest that functionalization of hydrogel matrices with appropriate binding sites remains a common strategy to modulate the release of highly hydrophilic cargos.^26^ Glycosaminoglycans (GAGs) have been described as beneficial moieties for the delivery of multiple growth factors and cytokines. GAGs constitute highly negatively charged polysaccharides capable of establishing electrostatic interactions with positively charged growth factors.^27^ Among different glycosaminoglycans, heparin remains highly interesting due to its high number of negative charges and its affinity to interact with different growth factors via specific binding sequences on its structure.^28^ Electrostatic interactions between heparin and several growth factors allow one to preserve these cargos attached to the biomaterial structure and, subsequently, allow its gradual release without influencing or modifying their protein structure and functionality, since they remain important modulatory signals in regenerative processes.^29^

In this work, a locally injectable iEDDA-cross-linked hydrogel is proposed for the encapsulation and controlled release of AEC-CM as an immunomodulatory and antifibrotic therapeutic alternative for tissue regeneration. An iEDDA-cross-linked injectable hydrogel formulation based on polyethylene glycol-tetrazine (PEG-TZ) functionalized with heparin (HEP) and hyaluronic acid-norbornene (HA-NB) was developed for the encapsulation of AEC-CM. The physicochemical, rheological, and swelling properties of the formulation were studied, as well as its applicability for the delivery and biological effect on immune cells of AEC-CM. *In vitro* release studies of the significant protein components of AEC-CM were performed over time. Moreover, *in vitro* biological studies were performed to study the immunomodulatory potential of the system. Therefore, the proliferation of peripheral blood mononuclear cells (PBMC) and the macrophages phenotypic polarization were studied. This approach developed here is expected to provide an advanced therapeutic strategy in regenerative medicine, with potential applications in various tissue types.

## Materials and Methods

### Materials

4-Arm 20 kDa polyethylene glycol-amine (PEG-amine, Creative PEGWorks, USA), 5-[4-(1,2,4,5-tetrazin-3-yl)benzylamino]-5-oxopentanoic acid (Tz-COOH, Sigma-Aldrich, USA), 1-methyl-2-pyrrolidinone (NMP, Sigma-Aldrich, USA), triethylamine (Sigma-Aldrich, USA), N,N,N′,N′-tetramethyl-O-(1H-benzotriazol-1-yl)uranium hexafluorophosphate (HBTU, Sigma-Aldrich, USA), diethyl ether (Sigma-Aldrich, USA), 20 kDa hyaluronic acid (HA, Creative PEGWorks, USA), a mixture of endo and exo with the predominant endo form of 5-norbornene-2-carboxylic acid (Sigma-Aldrich, USA), N,N-dimethylformamide (DMF, Sigma-Aldrich, USA), diisopropylcarbodiimide (Sigma-Aldrich, USA), deuterated dimethyl sulfoxide-d_6_ (DMSO, Sigma-Aldrich, USA), deuterium oxide (Sigma-Aldrich, USA), cysteine (Cys, Sigma-Aldrich, USA), lithium phenyl (2,4,6-trimethylbenzoyl)phosphinate (LAP, Sigma-Aldrich, USA), Ellman’s reagent (Sigma-Aldrich, USA), ethylenediaminetetraacetic acid (EDTA, Sigma-Aldrich, USA), heparin sodium salt (Sigma-Aldrich, USA), N-(3-(dimethylamino)propyl)-N′-ethylcarbodiimide (EDC, Sigma-Aldrich, USA), N-hydroxysuccinimide (NHS, Sigma-Aldrich, USA), phosphate-buffered saline (PBS, Gibco, USA), trypsin (Cytiva, HyClone, USA), fetal bovine serum (FBS, Gibco, USA), minimum essential medium eagle (α-MEM, Gibco, USA), l-glutamine (Gibco, USA), 1% amphotericin B (Gibco, USA), and 1% penicillin/streptomycin (PEST, Gibco, USA), RPMI-1640 (ThermoFisher Scientific, USA) were used.

#### PEG-Tetrazine and HA-Norbornene Synthesis

4-Arm 20 kDa PEG-amine was functionalized with Tz-COOH to prepare 4-arm PEG-tetrazine (PEG-TZ), following the previously described protocols.^30,31^ Briefly, 100 mg of 4-arm 20 kDa PEG-amine (0.005 mmol which corresponded to 0.02 mmol −NH_2_) was dissolved in 5 mL of NMP and mixed with triethylamine (2× to −NH_2_, 0.04 mmol) in a dry and argon-purged round-bottomed flask under an argon atmosphere for 15 min. Separately, Tz-COOH (2.5× to −NH_2_, 0.05 mmol) was dissolved in NMP and reacted with HBTU (2.5× to −NH_2_, 0.05 mmol) under an argon atmosphere for 5 min. Subsequently, the activated Tz-COOH was added to the initial PEG-amine mixture, and the functionalization was conducted for 15 h at room temperature under an argon atmosphere. The obtained product was precipitated in 10-fold volume excess of cold diethyl ether (−20 °C), centrifuged, dried overnight under vacuum, dialyzed against ultrapure water for 48 h, and lyophilized. PEG-TZ was additionally functionalized with heparin (PEG-TZ-HEP), following the previously described protocol.^32^ Briefly, heparin was mixed with EDC and NHS in Milli-Q water following a 2:1 molar ratio between EDC and NHS and a 5:1 molar excess of EDC in comparison to the amount of heparin carboxylic groups. The mixture was kept on ice (4 °C) for 15 min to activate the heparin carboxylic groups. Subsequently, PEG-TZ was dissolved in Milli-Q water on ice and added to the previously activated heparin solution following a molar ratio of 0.25:1 heparin to PEG-TZ. The functionalization was conducted overnight at 4 °C, and the obtained product was dialyzed against ultrapure water for 48 h and lyophilized.

HA was functionalized with 5-norbornene-2-carboxylic acid to obtain HA-norbornene (HA-NB), following previously described protocols with some modifications.^33^ First, 1 g of HA (0.05 mmol, which corresponds to 0.2 mmol of −OH) was dissolved in 10 mL of deionized water at room temperature under constant stirring. After its complete dissolution, 5 mL of DMF was slowly added, and the pH of the solution was adjusted to 10 by 5 M NaOH solution. In another dry and argon purged vessel, 5-norbornene-2-carboxylic acid (1:1 to −OH) was dissolved in 5 mL of DMF and mixed with diisopropylcarbodiimide (0.5:1 M to 5-norbornene-2-carboxylic acid) during 1 h at room temperature under an argon atmosphere to obtain dinorbornene anhydride. The obtained product was filtered to remove the resulting urea salts. Subsequently, dinorbornene anhydride was added dropwise to the HA solution, maintaining the pH of the solution at 10. The functionalization was conducted overnight at 4 °C. The obtained product was three times precipitated in 10-fold volume excess of cold ethanol-DMF (1/1 v/v) and centrifuged. Afterward, the product was dialyzed against ultrapure water for 48 h and lyophilized.

Both obtained polymers, PEG-TZ and HA-NB, were dissolved in deuterated DMSO and deuterium oxide for their characterization by H NMR to study the functionalization performed with an Ascend 400 MHz NMR spectrometer (Bruker, Switzerland). The functionalization degree of PEG-TZ was calculated based on the area associated with specific protons of the added moieties. The functionalization degree of HA-NB was studied following the previously described photoactivated thiol–ene reaction.^34^ First, predetermined concentrations of 5-norbornene-2-carboxylic acid and HA-NB were separately prepared in a buffer solution containing 10 mM Cys and 0.1% w/v LAP. Subsequently, the solutions were irradiated for 10 min with blue light (405 nm, 25 mW/cm^2^). Separately, predetermined concentrations of Cys were prepared in a 0.1 M sodium phosphate 1 mM EDTA reaction buffer, pH 8.0. After irradiation, the previous samples were diluted 10 times in a reaction buffer. Finally, all the samples were reacted with Ellman’s reagent, and absorbance at 412 nm was measured to determine the residual nonreacted Cys concentration. The concentration of norbornene in HA-NB was calculated based on the standard curve, and the degree of functionalization of HA-NB was calculated according to eq 1:
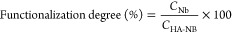
1where *C*_Nb_ is the concentration of norbornene group in HA-NB determined from NB standard curve, and *C*_HA-NB_ is the concentration of functionable groups in HA-NB (n = 3).

### Fabrication of HA-NB Hydrogels

HA-NB/PEG-TZ (HA-PEG) and HA-NB/PEG-TZ-HEP (HA-PEG-HEP) hydrogels were prepared at different concentrations of both polymers, maintaining a 1:1 ratio between the two reactive groups following the previously estimated modification degrees. Each polymer was dissolved in Milli-Q water, and subsequently, both precursor polymers were briefly mixed and left to cross-link for a few minutes.

### Morphological and Physicochemical Characterization: SEM, FTIR

The external and inner structures of PEG-TZ HA-NB hydrogels were characterized using scanning electron microscopy (SEM Quanta FEG 250, FEI, USA) at an accelerating voltage of 5.0 kV. The inner structure of the hydrogels was studied after the preparation of 15 μm thin segments (Leica Cryostat CM1950, Leica Biosystems, Germany). Samples were previously immersed in a cryostat embedding medium and frozen in liquid nitrogen. After sectioning the samples, segments were placed over a silica substrate, dried overnight under vacuum at room temperature, and coated with a 10 nm platinum layer.

PEG-TZ HA-NB hydrogels were studied by attenuated total reflectance Fourier transform infrared (ATR–FTIR) spectroscopy (Bruker Vertex 70, Bruker, USA). Dried hydrogels were studied in the range of 5000–500 cm^–1^ with a resolution of 2 cm^–1^, and FTIR spectra were recorded by using OPUS 8.1 software.

### Rheology Studies

Rheological studies were performed in HA-PEG and HA-PEG-HEP hydrogel samples using a TA Instruments DHR-2 rheometer (TA Instruments, USA), with a 20 mm diameter stainless steel plate-and-plate geometry (parallel plate). Gels were formed, immersed in PBS at 37 °C overnight, and loaded into the lower plate. Subsequently, strain sweep measurements were performed by applying a gradual strain variation from 0.1% to 1000% maintaining temperature and frequency values at 25 °C and 1 Hz. Subsequently, frequency sweep measurements were conducted modifying the applied oscillatory frequency from 20 to 0.1 rad/s. Meanwhile, the strain was maintained at 1% following the linear viscoelastic range, and the temperature was maintained at 25 °C. Moreover, time sweep measurements were conducted with both precursor polymer solutions quickly mixed and injected above placed on the lower plate, allowing the gel to cure. Therefore, the variation of *G*′ and *G*′′ was monitored under constant strain, angular frequency, and temperature values of 1%, 1 Hz, and 25 °C, respectively.

### Swelling Studies

The swelling index of the hydrogels was determined after immersing dried hydrogels in 1× phosphate buffer solution (PBS, pH 7.4) at 37 °C. The swelling index was calculated at specific time points following two different methodologies, either by determining the diameter of disc-shaped hydrogels using optical microscopy (Nikon SMZ645, Japan) and software analysis (ImageJ software) and subsequent calculation of the hydrogel volume or by weighing hydrogels after carefully removing the excess of water on their surface. At each time point, the swelling index was calculated based on the volume following eq 2:
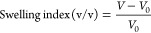
2where *V* corresponds to the volume of swollen hydrogels at a particular time point, and *V*_0_ corresponds to the volume of the dried hydrogels. Moreover, the swelling index was also calculated based on the weight following eq 3:
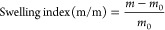
3where *m* corresponds to the volume of swollen hydrogels at a particular time point, and *m*_0_ corresponds to the volume of the dried hydrogels.

### Isolation and Culture of Ovine Amniotic Epithelial Cells

AEC were isolated from the amniotic membrane (AM) of three pregnant Appenninica sheep during midgestation (when fetuses measured 25–30 cm) to prevent the epithelial-mesenchymal transition (EMT) typically occurring at term stage. The uterus wall was carefully opened to obtain sterile amnios, and the AM was separated from the chorion by using sterile tweezers under a stereomicroscope. The AM tissue was then cut into 2–3 cm fragments and washed in calcium- and magnesium-free PBS. AEC were extracted by incubating the fragments in 0.25% Trypsin-EDTA at 38.5 °C for 40 min with gentle agitation. The enzymatic reaction was then stopped by adding 10% FBS, and the solution was filtered through a 40 μm membrane. Cells were collected by centrifugation at 500*g* for 10 min, and viable cells were counted using a LUNA-II Automated cell counter and trypan blue staining. AEC were then cultured in 6-well plates at a density of 100,000 cells per well in α-MEM, supplemented with 10% inactivated FBS, 1% l-glutamine, 1% amphotericin B, and 1% penicillin/streptomycin, along with 25 μM progesterone (P4) to prevent EMT. The cells were incubated at 38.5 °C with 5% CO_2_ until they reached 70% confluence, after which they were trypsinized with 0.05% Trypsin-EDTA for further experiments.

### Secretome Production

The production of the secretome began once freshly isolated AEC (P0) reached 70% confluence in the 6-well plates. The cells were then preconditioned in a serum-free medium (αMEM with 1% penicillin/streptomycin, 1% amphotericin B, and 1% l-glutamine) for 4 h. Based on previous studies involving other cellular models and AEC, cells were exposed to an inflammatory stimulus using LPS (1 μg/mL) for 1 h.^35^ After this treatment, all AEC groups (both control and LPS-treated) were washed twice with PBS and cultured in a serum-free medium for 24 h. Afterward, the secretome was collected and centrifuged at 500*g* for 10 min to remove cell debris. The supernatant was collected and stored at −80 °C until further use.

### Secretome Lyophilization

AEC-CM was lyophilized using the freeze-drying apparatus (VirTis BenchTop 2.0, SP Scientific, Gardiner, NY, USA). First, 5% trehalose was added to AEC CM as cryoprotectant before freezing the CM at −80 °C. Subsequently, AEC CM was lyophilized under vacuum conditions at <1 mbar of pressure and −110 °C for 24 h. Afterward, AEC CM was stored at −80 °C.

### Secretome Release Studies

AEC secretome-loaded hydrogels were prepared according to a previously described protocol for the preparation of PEG-TZ HA-NB hydrogels. Briefly, 1 month-old freeze-dried AEC secretome was concentrated 10x and dissolved in both precursor polymer solutions.

*In vitro* release studies were performed in 1x PBS. AEC secretome-loaded hydrogels were immersed in 5 mL of 1× PBS at 37 °C. The complete release medium was removed at specific time points, from 5 min to 14 days. After the complete removal of release medium at each time point, an equal volume of fresh buffer was immediately added. Subsequently, the total protein concentration at each time point was determined using a micro-BCA assay (Thermo Fisher, USA). Moreover, vascular endothelial growth factor (VEGF) and amphiregulin (AREG) concentrations were also determined at each time point using ELISA (Thermo Fisher, USA) and (#MBS044705; MyBiosource, Southern California, San Diego, USA), respectively, following manufacturer’s instructions. Afterward, the obtained data were analyzed following different kinetic models including zero-order, first-order, Higuchi, and Korsmeyer–Peppas models as described in eqs 4–8:
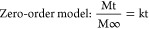
4
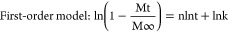
5
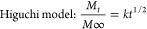
6
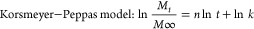
7
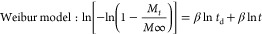
8where *M*_*t*_*/M*_*∞*_ corresponds to the proportion of protein released at time *t*; *k* corresponds to the corresponding release constant according to each specific model; *n* corresponds to the release exponent; *t*_d_ corresponds to the lag time before the drug release; β corresponds to a constant describing the shape of the release curve.

### PBMC Activation Assay

The immunomodulatory properties of AEC were evaluated by analyzing the proliferation of peripheral blood mononuclear cells (PBMC). Ovine PBMC were isolated using density gradient centrifugation with 12 mL of Ficoll-Paque PLUS (#GE17-1440-02; Cytiva, Marlborough, MA, USA) and 16 mL of peripheral blood, following the manufacturer’s instructions. The PBMC were activated with phytohemagglutinin (PHA) at a final concentration of 10 μg/mL. A total of 200,000 PHA-stimulated PBMC were then plated and cultured for 72 h and 7 days with the secretome derived from immune-activated AEC (stimulated with LPS) and encapsulated within PEG-TZ-HEP HA-NB hydrogels. PBMC proliferation was measured using the CellTiter96 Aqueous One Solution Cell Proliferation Assay according to the manufacturer’s instructions.

### Macrophage Polarization Studies

The immunomodulatory properties of AEC were further evaluated by assessing the macrophage M1 polarization toward the M2. Human PBMCs derived from three healthy patients were isolated by density gradient centrifugation in a Ficoll-Paque PLUS (GE Healthcare). The PBMCs were washed once in PBS and resuspended in RPMI-1640 with 10% FBS. For monocyte isolation, 6 × 10^6^ PBMCs were plated in each Nuclon Delta surface treated 35 mm Petri dishes (ThermoFisher Scientific) at 3 × 10^6^ to PBMCs/mL in 2 mL and allowed to adhere in a 5% CO_2_ container at 37° for 2 h. Nonadherent cells were removed by thorough washing with RPMI-1640. Adherent cells were harvested after incubation in Accutase for 15 min (561527, BD Biosciences, USA). For macrophage differentiation, cells were then centrifuged at 350*g* for 5 min and cultured in nontreated 12 well plates at a cell density of 3 × 10^5^ cells/mL with macrophage serum free medium (SFM) + 10% FBS + 1% penicillin/streptomycin enriched with 100 ng/mL of granulocyte-macrophage colony stimulating factor (GM-CSF) for 5 days. Cells were then stimulated with either 100 ng/mL LPS and 20 ng/mL IFN-γ (M1-stimulation), 40 ng/mL IL-4 and 40 ng/mL IL-13 (M2 stimulation), or remained untreated for 48 h. Furthermore, without removing the stimulus, AEC secretome-loaded hydrogels were added to M1 macrophages with a transwell set up (3401, Corning Incorporated, USA). After 48 h of treatment, the macrophages were harvested for flow cytometry analysis by incubating in Accutase (561527, BD Biosciences, USA) for 15 min. Cells were transferred to a V bottom 96-well plated and centrifuged at 350*g* for 5 min. The supernatant was discarded, and the macrophages were resuspended in FC block solution (10% TruStain FcXTM, BioLegend) in PBS) and incubated for 10 min at room temperature. Then, the cell pellets were immunostained with APC anti-CD86 and PE anti-CD206 antibodies at a concentration of 2 μg/mL in PBS at 4 °C for 30 min in the dark. Following this, the cells were washed twice with PBS and analyzed by using flow cytometry. For each group, cells without antibody staining served as the negative control. The fold change of the mean fluorescence intensity (MFI) in each sample was calculated by subtracting the MFI of the unstained samples and normalizing against the negative control. All flow cytometry data were analyzed by using FlowJo software.

### Statistical Analysis

Quantitative data were obtained by analyzing each sample in triplicate, with each experimental condition replicated at least three times. The results were initially assessed for distribution using the Shapiro–Wilk test and then analyzed using a *t* test or one-way ANOVA, followed by Tukey’s post hoc test (GraphPad Prism 10, GraphPad Software, San Diego, CA, USA). Significance was set at *p* < 0.05.

### Ethical Statement

Patient’s participation and sample collection were obtained after receipt of a signed informed consent following the ethical permit number 3/2023 granted by the Finnish red cross. No ethic statement is required for the present research since Ovine AEC has been isolated from waste biological samples collected at the abattoir from animals slaughtered for food purposes.

## Results and Discussion

### PEG-Tetrazine and HA-Norbornene Synthesis and Hydrogel Characterization

4-Arm PEG-TZ and HA-NB, both precursor polymers, were functionalized following different procedures previously described in the literature.^30^ For the preparation of PEG-TZ, 4-arm PEG was coupled with Tz-COOH following a highly common acid-amine conjugation approach. Moreover, HA-NB was prepared by activation of the hydroxides from HA, which therefore acted as nucleophiles against dinorbornene anhydride. Following functionalization, the structures of both modified precursors were studied by ^1^H NMR (Figures S1–S2). The obtained PEG-TZ spectra showed the characteristic peaks at δ 10.5, 8, and 7.5 ppm previously correlated with the tetrazine group protons and a functionalization degree of approximately 75%.^36^ Moreover, the obtained HA-NB spectra also showed the presence of specific peaks at δ 6 and 6.5 ppm corresponding to norbornene protons.^37^ The functionalization degree was determined by the obtained ^1^H NMR spectra and the previously described thiol-norbornene reaction, which confirmed that the substitution degree of HA-NB was approximately 30% based on the average obtained by both methods (Figure S3).

**Figure 1:**
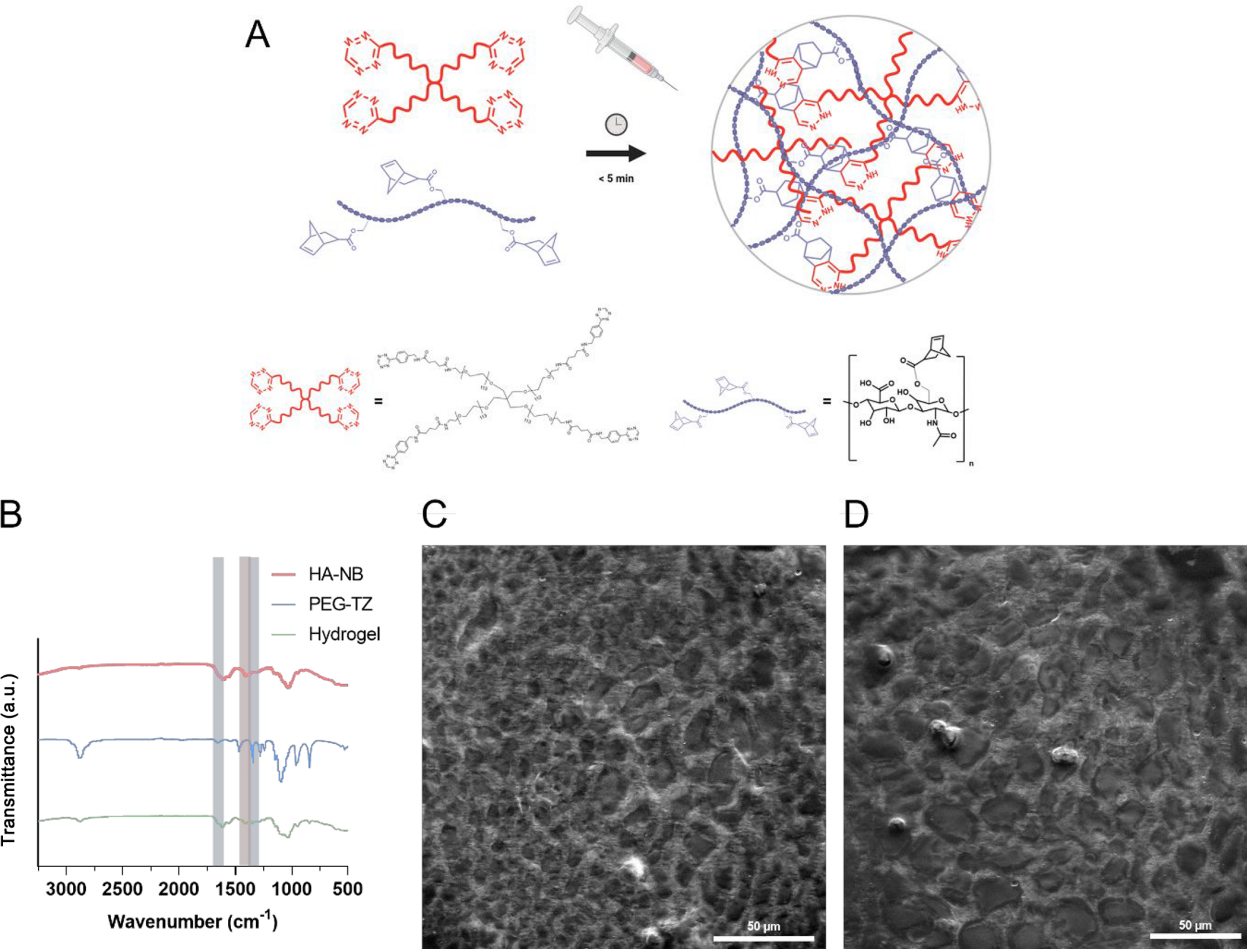
PEG-tetrazine and HA-norbornene synthesis and hydrogel production. (A) Schematic representation of the development and structure of PEG-HA TZ-NB cross-linked hydrogel. (B) ATR–FTIR spectra of HA-NB, 4-arm PEG-TZ, and HA-PEG hydrogel. FTIR spectra were recorded within the 3500–500 cm^–1^ range with a resolution of 2 cm^–1^. (C) SEM image of the inner structure of the HA-PEG hydrogel. Scale bar: 50 μm. (D) SEM image of the inner structure of the HA-PEG-HEP hydrogel. Scale bar 50 μm.

Subsequently, the PEG-HA hydrogel was produced by simply mixing both precursor polymers in solution at room temperature (Figure 1A). The composition of the hydrogel formulation maintained a 1:1 ratio between both tetrazine and norbornene groups, with a final HA-NB concentration of 12.5% w/v.

Initial physicochemical characterization studies were conducted using ATR–FTIR. Following Figure 1B, FTIR analysis confirmed the substitution of HA-NB by the observation of a characteristic band at 1410 cm^–1^ corresponding to the C=C bond from norbornene.^21^ PEG-TZ spectrum showed pronounced bands at 1630 and 1360 cm^–1^ corresponding to the tetrazine double bonds between C=N and N=N, respectively. Furthermore, characteristic bands correlated with the structure of the functionalized polymer were also observed at 1100 and 2880 cm^–1^, corresponding to the C–O and C–H stretching, respectively.^38^ The formed hydrogel spectrum did not show either any band at 1630 or 1360 cm^–1^, demonstrating that the reaction between both functional groups was completed, and the C=N and N=N bonds were no longer part of the cross-linked structure, resulting in the released gaseous nitrogen.

Morphological characterization studies were conducted using SEM. The obtained images from the inner structures of both HA-PEG and HA-PEG-HEP hydrogel formulations correlated with highly porous polymer structures and did not show significant differences between both formulations. The images indicated similar structures as other similar hydrogel formulations with the used precursor polymers and cross-linking method, which is responsible for the release of gaseous nitrogen as cross-linking byproduct and the formation of porous matrices.^39^

**Figure 2:**
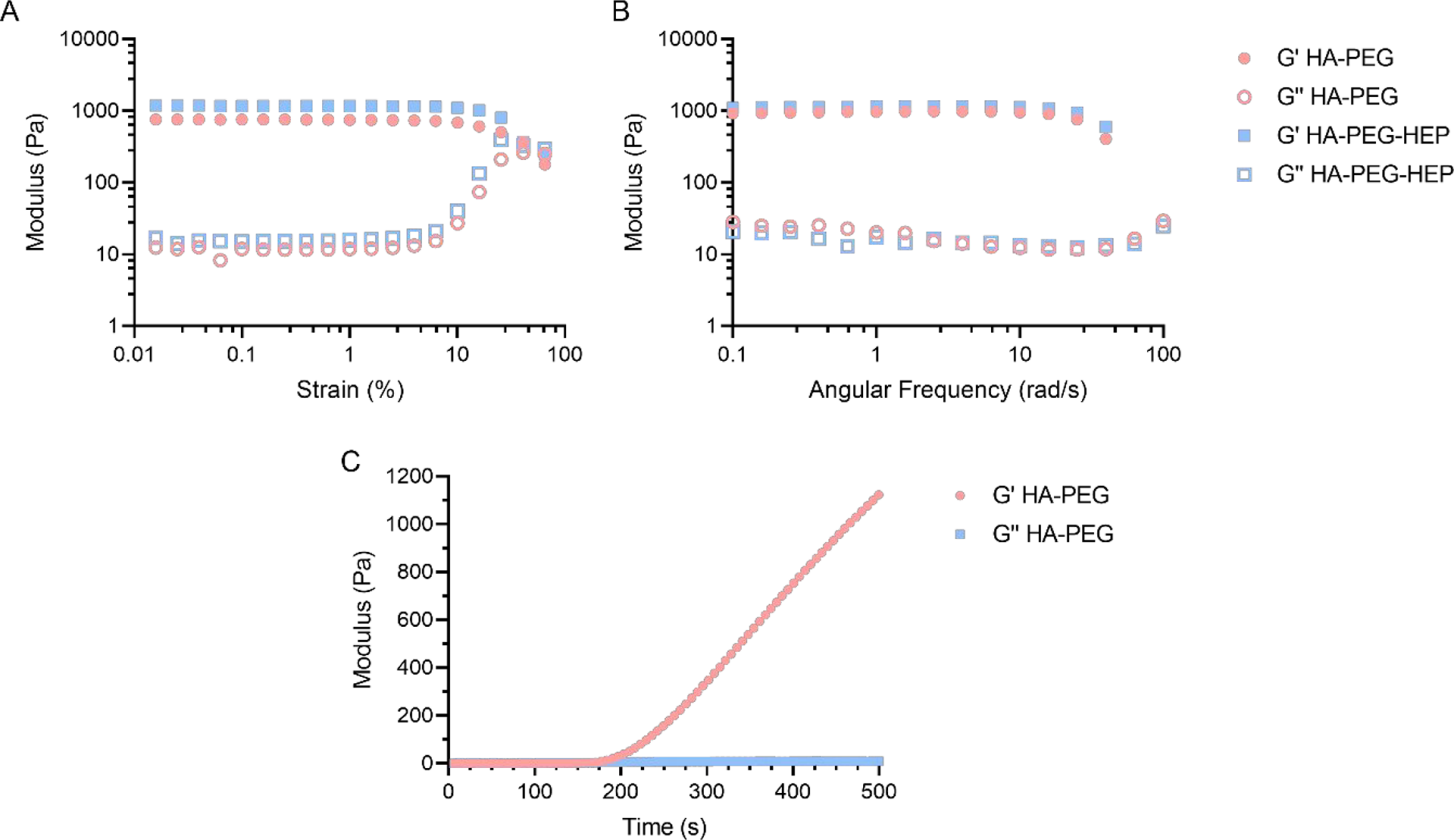
Rheological studies in HA-PEG and HA-PEG-HEP hydrogels. (A) Strain sweep measurements of HA-PEG and HA-PEG-HEP. (B) Frequency sweep measurements of HA-PEG and HA-PEG-HEP. (C) Time sweep measurements of HA-PEG and HA-PEG-HEP.

Subsequently, the rheological characterization of HA-PEG and HA-PEG-HEP hydrogels was performed. Both types of formulations were produced with a disc-like morphology and immersed in 1× PBS during 24 h. Hydrogels were characterized by strain sweep and frequency strain measurements. Moreover, time sweep measurements were performed immediately after mixing both precursor polymer solutions to study the gelation kinetics of the system. Figure 2 shows that strain sweep measurements of both types of hydrogels owned almost identical *G*′ and *G*′′ profiles, which showed a highly stable trend until 10% strain. Therefore, frequency sweep measurements were conducted within the previously observed linear viscoelastic range. HA-PEG and HA-PEG-HEP showed again identical behavior, with constant *G*′ values up to 930 and 1110 Pa, respectively. It has been extensively described that the combination of HA with other synthetic polymers is usually conducted with the aim to improve the rheological properties of standard HA-based hydrogels. The obtained results indicate that both hydrogel formulations showed similar *G*′ and *G*′′ values in comparison with other HA-PEG hydrogel formulations with a similar initial polymer concentration.^40^

Time sweep measurements of the precursor polymers indicated that the developed HA-PEG formulation experienced a *G*′ crossover with *G*′′ after approximately 200 ± 84 s following the mixing of both polymers, corresponding to the initial gelation time. Subsequently, *G*′ values continued increasing up until complete gelation, which was detected shortly after 500 s. The observed behavior remains similar to other hydrogel systems with the same cross-linking principle and comparable concentration of precursor polymers.^41^

### Swelling Studies

After the physicochemical and mechanical properties were investigated, the swelling properties of the developed PEG-HA hydrogel were assessed with the aim to acquire a more in-depth knowledge of the water absorption capabilities of the system. The swelling behavior of both PEG-HA and HA-PEG-HEP formulations was studied following either volume or weight changes after immersing dry hydrogels in 1× PBS at 37 °C. At specific time points, the diameter of disc-shaped hydrogels was measured to calculate the volume of each sample. Moreover, hydrogels were also weighed after removal of the excess PBS. In each case, both volume and mass were compared to their respective initial dry values before immersing hydrogels in PBS.

**Figure 3:**
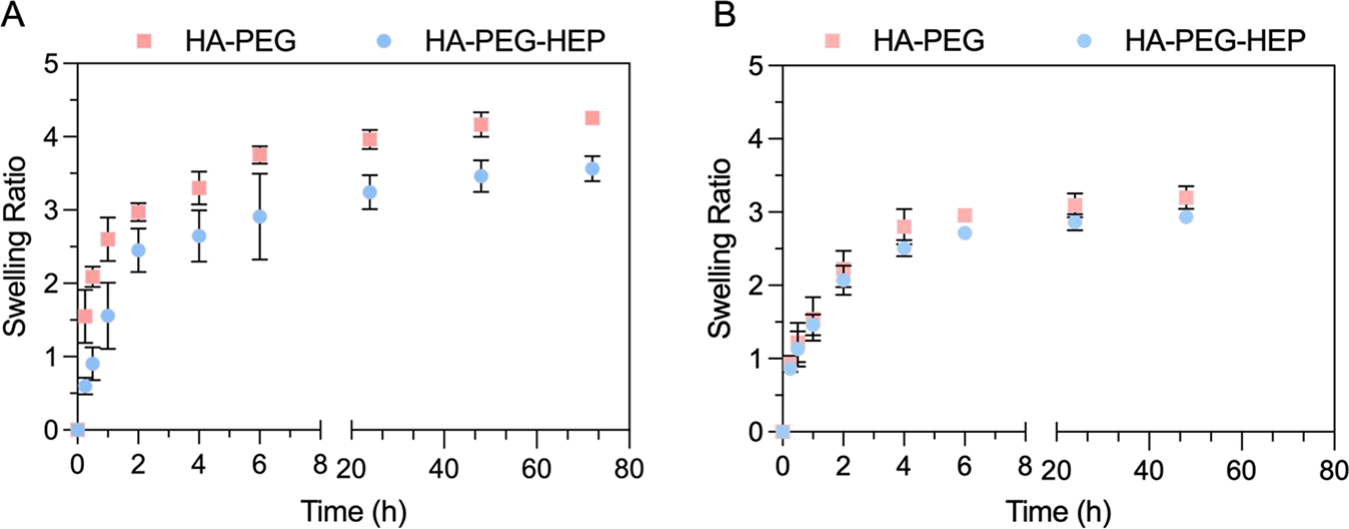
Swelling studies in HA-PEG and HA-PEG-HEP hydrogels. (A) Swelling ratio calculated based on the measured volume of HA-PEG and HA-PEG-HEP hydrogels incubated in 1× PBS at 37 °C. (B) Swelling ratio calculated based on the mass of HA-PEG and HA-PEG-HEP hydrogels incubated in 1× PBS at 37 °C. Results are presented as mean ± SD (*n* ≥ 3).

Figure 3 shows an initial swelling ratio increase during the early 2 h following immersion of both types of hydrogels. After 2 h, volume-based measurements indicated that PEG-HA and PEG-HA-HEP hydrogels showed swelling ratio values of 2.9 and 2.4, respectively. Subsequently, the swelling ratio of both formulations showed a limited and constant increase after 24 h, following a stable evolution after 48 and 72 h, with PEG-HA and HA-PEG-HEP swelling ratios of 4 and 3.5, respectively. Mass-based measurements indicated initial swelling ratio values of 2.2 for both types of formulations after 2 h, followed by a reduced increase up to 3 in both types of hydrogels after 24 h. HA and PEG-based hydrogels have previously been reported to have considerably high swelling ratio values due to the hydrophilic properties of both polymers.^42,43^ The swelling properties can be conditioned by a wide variety of factors, including the degree of cross-linking, the presence of additional moieties in the polymer network, or the loading of cargos. Generally, higher cross-linking degree or polymer modification degree are correlated with lower swelling ratios.^44^ The developed PEG-HA and PEG-HA-HEP hydrogels show reduced swelling degree values in comparison with other PEG and HA-based hydrogel formulations with different cross-linking methods.^45^ The observed reduction in the swelling degree can be explained by the higher cross-linking density achieved by the specific iEDDA cross-linking method of the developed system. In fact, studies conducted in other iEDDA-cross-linked hydrogels show a similar tendency to own lower swelling properties in comparison with other cross-linking methods.

Moreover, the developed HA-PEG and HA-PEG-HEP formulations showed similar swelling properties. Despite mass-based measurements not showing any differences between the swelling behavior of both types of formulations, volume-based measurements indicated that HA-PEG-HEP exhibited slightly lower swelling ratio values. The observed differences between the two types of studies were not found to be significant and may be attributed to the variability in the methods used. Nevertheless, both formulations demonstrated relatively low swelling properties, which is advantageous for maintaining structural integrity and controlling the release of encapsulated agents over time.^46^ The swelling behavior of hydrogels is intricately linked to their degradation profile, as both phenomena are governed by the hydrogel’s cross-linking density and the chemical structure of the polymer network.^47^ In general, hydrogels with higher swelling ratios tend to degrade more rapidly, because the increased water absorption facilitates hydrolytic or enzymatic cleavage of the polymer chains. Conversely, lower swelling ratios, as observed in the PEG-HA and PEG-HA-HEP hydrogels in this study, often correlate with a more stable network that degrades more slowly.^47,48^ This relationship suggests that the observed swelling profile of these hydrogels, characterized by a limited and stable increase over time, may indicate a slower degradation rate.

### Secretome Release Studies

To study the effect of incorporating heparin as an additional moiety, the release kinetics of AEC-CM loaded HA-PEG-HEP and HA-PEG hydrogel formulations were studied. Both types of formulations were prepared and loaded with lyophilized AEC-CM, immersed in 1× PBS at 37 °C, and the complete release medium was removed and replaced at specific time points. Subsequently, the total protein concentration from the sample obtained at each point was quantified, as well as the concentration of two specific proteins previously reported as predominant components of AEC-CM, VEGF^49^ and AREG,^50^ as two model proteins.

**Figure 4:**
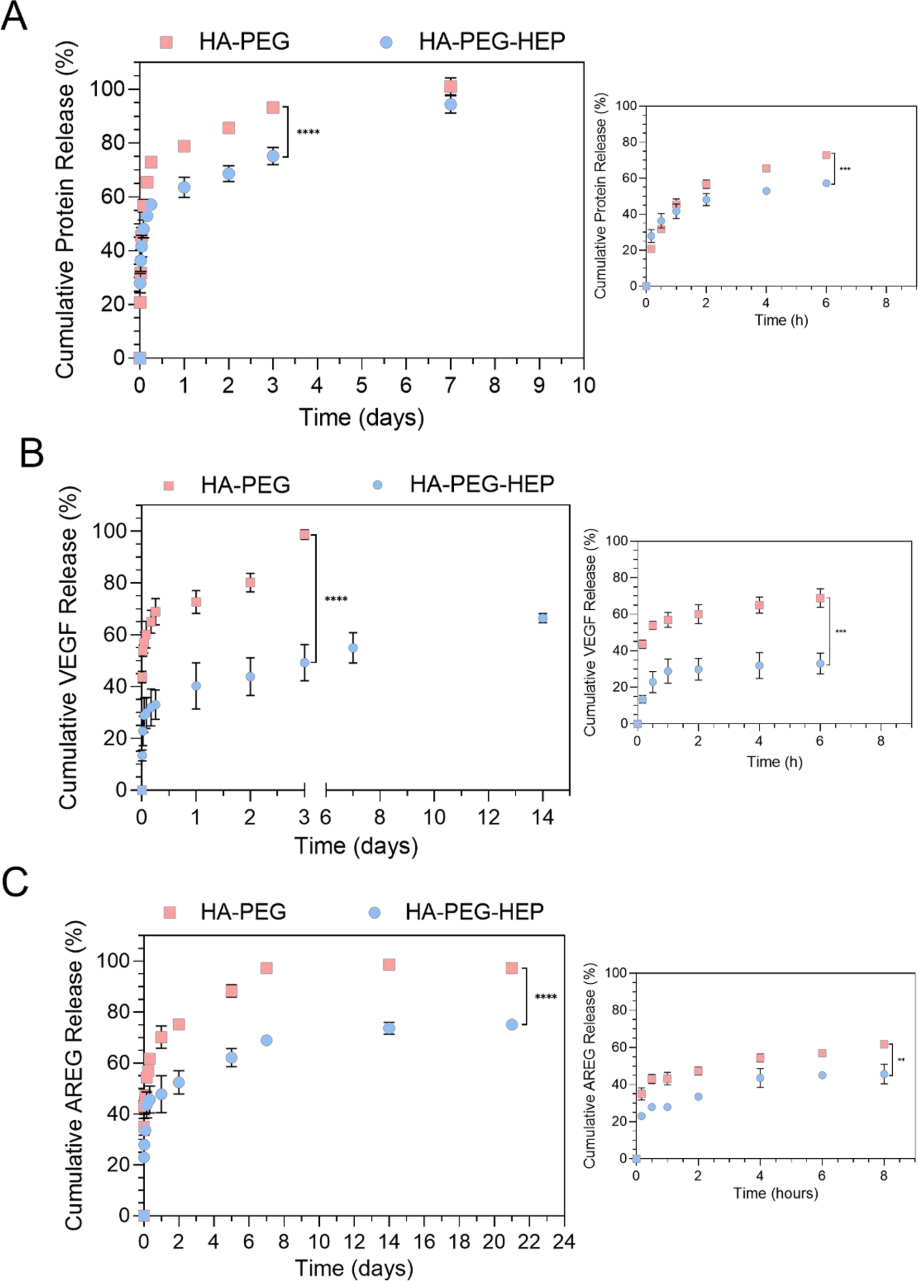
Cumulative release studies in AEC-CM loaded HA-PEG and HA-PEG-HEP hydrogels. (A) Cumulative total protein release profile from HA-PEG and HA-PEG-HEP hydrogels in 1× PBS at 37 °C for 21 days and the magnified cumulative release profile during the initial 6 h. (B) Cumulative VEGF release profile from HA-PEG and HA-PEG-HEP hydrogels in 1× PBS at 37 °C for 21 days and the magnified cumulative release profile during the initial 6 h. (C) Cumulative AREG release profile from HA-PEG and HA-PEG-HEP hydrogels in 1× PBS at 37 °C for 21 days and the magnified cumulative release profile during the initial 6 h. Results are presented as mean ± SD (*n* ≥ 3), and the samples were analyzed with ordinary one-way ANOVA, followed by Turkey posthoc test, setting at the probabilities *p < 0.05, **p < 0.01, ***p< 0.001, ****p< 0.0001 comparing each HA-PEG and HA-PEG-HEP sample within the same time point.

As shown in Figure 4A, the total protein content and the two specific proteins showed an initial burst release during the earliest hours of the study, as it has been previously correlated with highly hydrophilic cargos.^51−54^ However, the obtained results indicated significant differences, depending on the type of formulation and the studied cargo. Total protein studies showed burst releases of 50 and 60% of the total protein content from HA-PEG-HEP and HA-PEG formulations, respectively. After 24 h, HA-PEG-HEP and HA-PEG hydrogels had released around 60 and 80% of the total protein content, followed by a sustained release of the remaining content up to 7 days in HA-PEG and 14 days in HA-PEG-HEP. The VEGF release profile showed an initial burst release of approximately 30 and 60% in HA-PEG-HEP and HA-PEG gels, respectively. The differences were maintained after 24 h, with HA-PEG-HEP and HA-PEG releasing 40 and 70%, respectively. Subsequently, the remaining VEGF was released at a sustained rate with highly significant differences. Even though HA-PEG gels released the complete amount of VEGF after 3 days, HA-PEG-HEP gels showed a much slower controlled release with only 60% of VEGF content released after 14 days. Additionally, AREG release studies also showed initial burst releases of 40 and 50% in HA-PEG-HEP and HA-PEG gels, respectively. Afterward, both formulations showed a sustained release, with HA-PEG gels releasing the total AREG content after 7 days and HA-PEG-HEP gels releasing up to 65% after 7 days and maintaining a slow-release trend for more than 21 days.

Burst release of hydrophilic cargos is frequently observed in hydrogel formulations due to their highly porous structure, which depends on the cross-linking degree and molecular weight of the precursor polymers, among other parameters. Considering the described limitations, functionalization of the polymer matrix with heparin was proposed. Heparin is a commonly used GAG with numerous sulfate groups and predominant negative charge, allowing one to stablish electrostatic interactions with positively charged cargos like growth factors, among other molecules.^55^ The obtained data showed that functionalization with heparin allowed us to reduce the burst release degree of the three monitored cargos. Moreover, the initial decrease in the percentage of burst released cargo in HA-PEG-HEP hydrogels was followed by a more prolonged release of the nonreleased cargo. The initial reduction was considerably limited for the total protein content but statistically significant for both VEGF and AREG. These differences may be explained by the presence of electrostatic interactions between heparin and different components from AEC-CM, reducing the initial burst release and slowing the process.

The study of release kinetics was performed to gain a better understanding of the observed processes. We analyzed the obtained data according to the zero-order, first-order, Higuchi, Korsmeyer–Peppas, and Weibull models. Table 1 shows that the highest correlation coefficient (R^2^) values for all of the formulations corresponded to both Korsmeyer–Peppas and Weibull models. The Korsmeyer–Peppas model has commonly been used to describe release kinetics despite its important limitations, since it can only be used for the data up to the 60% of the cumulative released cargo. The model can be used to predict the release mechanism of the studied system. Specifically, release exponents *n* ≤ 0.43 are correlated with Fickian diffusion behavior, *n* ≥ 0.85 correspond to controlled or polymer relaxation-mediated transport behavior, and 0.43 < *n* < 0.85 correspond to anomalous or non-Fickian release, combining both diffusion and polymer relaxation explanations.^56^ Moreover, the Weibull model is commonly used to predict and describe the release profiles, not the release kinetics of a system. It allows one to determine the shape of a release curve thanks to the shape parameter, β, which indicates an exponential release profile when β = 1, a sigmoidal profile when β > 1, and a parabolic profile when β < 1.^57^

**Table 1:** Release Kinetics Analysis^a^

		Correlation coefficient (r^2^)			Korsmeyer–Peppas model		Weibull model	
Formulation		Zero-order model	First-order model	Higuchi model	R^2^	n	R^2^	β
Total Protein	HA-PEG-Hep	0.580	0.566	0.827	0.992	0.198	0.931	0.262
	HA-PEG	0.521	0.310	0.704	0.993	0.378	0.971	0.371
VEGF	HA-PEG-Hep	0.608	0.514	0.799	0.932	0.173	0.948	0.219
	HA-PEG	0.496	0.753	0.636	0.944	0.128	0.778	0.246
AREG	HA-PEG-Hep	0.553	0.538	0.752	0.927	0.156	0.976	0.211
	HA-PEG	0.502	0.540	0.720	0.966	0.130	0.938	0.296

a Release studies were analyzed following zero-order, first-order, Higuchi, Korsmeyer–Peppa,s and Weibull models, and the parameters corresponding to each model were calculated.

All the released studies with the two types of formulations and the quantification of different types of cargo showed a similar pattern, with a good fit with the Korsmeyer–Peppas model and reduced exponent *n* values below 0.43. Therefore, the release of the three monitored cargos was defined by a Fickian diffusion mechanism, which may be explained by the high hydrophilic nature of the studied cargos, their high diffusion coefficient, and the highly porous hydrogel matrices. Moreover, the data also showed a particularly good fit with the Weibull model, and all the release profiles predominantly showed a parabolic shape, with β values below 1.

Overall, the two tested systems showed an initial diffusion-mediated burst release due to the high diffusion coefficient of the loaded cargo and the porous nature of the system. However, incorporation of heparin as an additional moiety in the hydrogel structure proved to reduce the percentage of initially burst released cargo and subsequently prolong the sustained release of the monitored cargos. This phenomenon can be explained by the negative charge of the functionalized hydrogel due to the high number of sulfate groups in the heparin structure, which can establish electrostatic interactions with positively charged cargos, including proteins such as the monitored two model molecules from AEC-CM. The incorporation of heparin proved to be an efficient method to achieve a more sustained release of the loaded AEC-CM and, most importantly, reduce the initial burst release. This sustained release is particularly beneficial in managing inflammatory conditions in host tissue, as it ensures a more controlled and prolonged therapeutic effect, thereby helping to modulate the inflammatory response over time.^12,13^ The observed tendency and the degree of burst release decrease correlate with previous studies conducted with specific growth factors or conditioned media from other source cells.^58−61^ Therefore, the HA-PEG-HEP hydrogel was selected as the optimal formulation to conduct the following *in vitro* biological efficacy studies.

### Immunomodulatory Enhancement of AEC Secretome-Loaded Hydrogels

**Figure 5:**
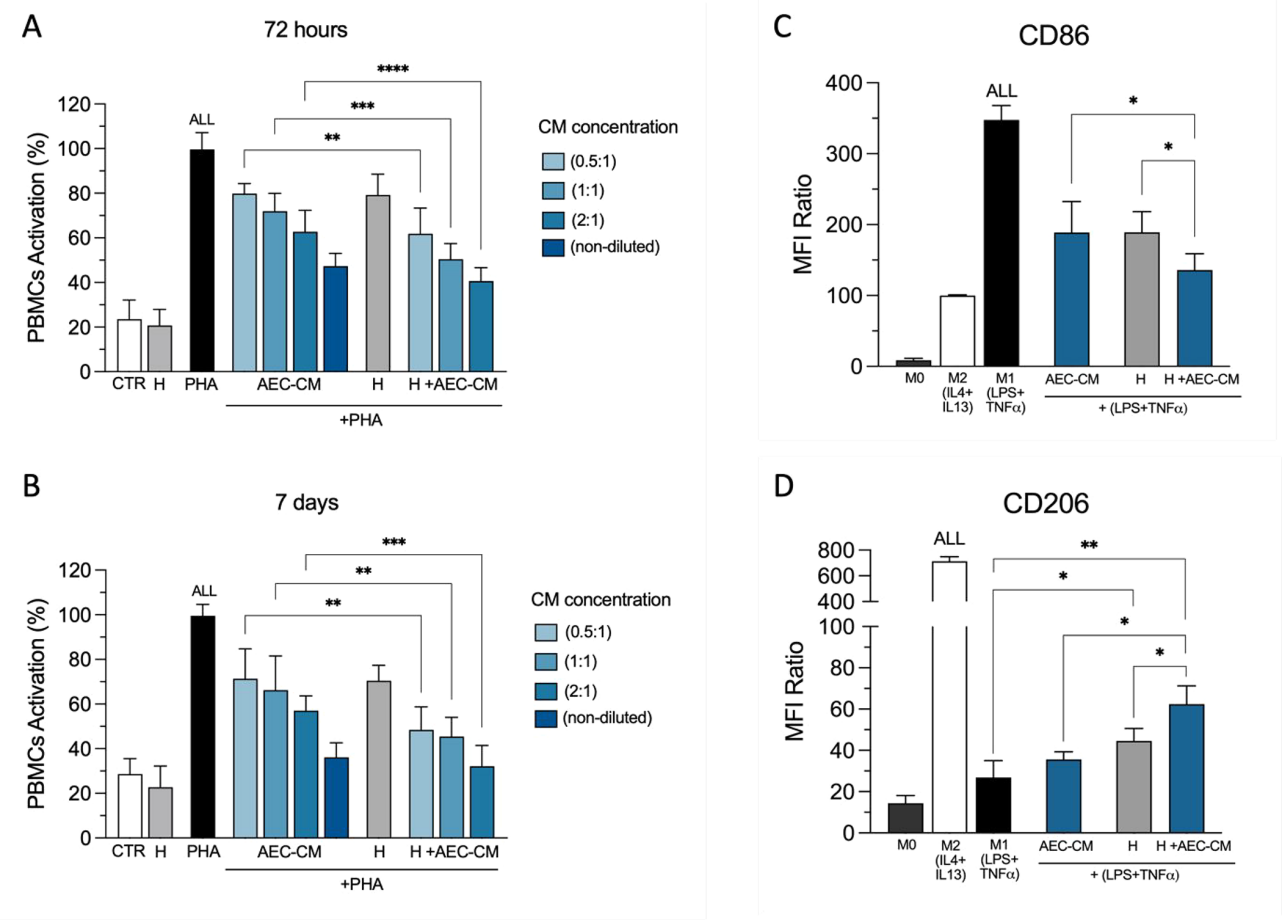
Immunomodulatory effect of hydrogel encapsulated AEC-CM. PBMCs activation after (A) 72 h and (B) 7 days of culture with PHA and treatment with different AEC-CM concentrations with or without the HA-PEG-HEP encapsulation (H + AEC-CM). (C) CD86 expression of human macrophages treated with AEC-CM alone, HA-PEG-HEP hydrogel alone (H), or the combination with the encapsulation (H + AEC-CM) for 48 h. (D) CD206 expression of human macrophages treated with AEC-CM alone, HA-PEG-HEP hydrogel alone (H), or the combination with the encapsulation (H + AEC-CM) for 48 h. Data (mean ± SD) represent three independent sets of experiments (*n* = at least three biological replicates in each group per set; each biological replicate assayed in at least three technical replicates). Statistically significant values between the different studied groups were obtained (*p < 0.05, **p < 0.01, ***p < 0.001, and ****p < 0.0001, respectively).

To assess the immunomodulatory properties of the AEC secretome-loaded hydrogels, a PBMC proliferation assay was conducted at 3 and 7 days to assess the early and midlate effect, once the phenotype of the AEC and the neglectable detrimental effect of the lyophilization in the secretome in terms of protein content and biological effect were confirmed (Figure S4). As shown in Figure 5A and B, the encapsulation of AEC-derived secretomes in the HA-PEG-HEP hydrogels significantly enhanced their immunomodulatory effects. At 3 days, the PBMC activation percentages for the AEC-CM groups decreased in a concentration-dependent manner, with higher concentrations (nondiluted) showing the most substantial suppression of around 60% (*p* < 0.001 vs. PHA, Figure 5A), which resemble the observations previously obtained in other studies.^50,62,63^ Interestingly, the hydrogel-encapsulated secretome suppressed PBMC activation more effectively than the nonencapsulated counterparts at all the concentrations [*p* < 0.01, H + AEC-CM (0.5:1) vs. AEC-CM (0.5:1); *p* < 0.001, H + AEC-CM (1:1) vs. AEC-CM (1:1); *p* < 0.0001, H + AEC-CM (2:1) vs. AEC-CM (2:1), Figure 5A]. Of note, the hydrogel alone (H) induced a slight immunosuppressive effect (*p* < 0.01 vs. PHA, Figure 5A).

At longer exposition times of 7 days, a more pronounced overall inhibition of PBMC activation was observed across all conditions (Figure 5B). In detail, the secretome-encapsulated hydrogel continued to exhibit a greater suppression of PBMC activation compared to the nonencapsulated secretome at all the concentrations. The PBMC activation percentages for the AEC-CM groups remained significantly lower than the positive control, particularly at higher concentrations (*p* < 0.01, H + AEC-CM (2:1) vs. PHA, Figure 5B). Similarly, the hydrogel alone (H) maintained its slight immunosuppressive effect (*p* < 0.01 vs. PHA, Figure 5B). These findings highlight the enhanced immunosuppressive capabilities of the AEC secretome when encapsulated in a hydrogel system and suggest a synergistic effect between the hydrogel and the AEC secretome.^64,65^ The obtained data also suggested that nonactivated PBMCs cultured with the empty HA-PEG hydrogel did not exhibit significant proliferation differences in comparison to the control group, proving that the developed system owned high cytocompatibility with PBMCs.

Building on the findings from the PBMC activation assay, the analysis of human macrophage polarization further underscored the enhanced immunomodulatory properties of the AEC secretome when it was encapsulated in the HA-PEG-HEP hydrogels. The use of human PBMCs to obtain M0 macrophages highlights the importance of xenocross talk between ovine-conditioned media and human immune cells, which may provide insights into the broader applicability of the findings across species. Additionally, the 48-h time point was chosen for its suitability in capturing early macrophage polarization dynamics, allowing for the observation of initial shifts in phenotype.^66^ As shown in Figure 5C and D, the analysis revealed that all tested conditions (the AEC-CM alone, hydrogel alone, and AEC-CM-loaded hydrogel) positively induced a shift in macrophage polarization from the pro-inflammatory M1 phenotype toward the anti-inflammatory M2 phenotype. This shift was evidenced by a reduction in the mean fluorescence intensity (MFI) for M1 marker CD86 (Figure 5C) and an increase in the MFI for M2 marker CD206 (Figure 5D).

Specifically, for the M1 marker CD86, the hydrogel-encapsulated secretome (H + AEC-CM) resulted in the most significant reduction in MFI, indicating the highest suppression of the M1 phenotype. The AEC-CM alone and the hydrogel alone also reduced CD86 MFI, but to a lesser extent compared to the encapsulated secretome (*p* < 0.05 vs. H + AEC-CM, Figure 5C). For the M2 marker CD206, similar results were obtained. The AEC-CM alone and the hydrogel alone increased CD206 MFI, but the effect was more substantial when the secretome was encapsulated within the hydrogel (*p* < 0.05 vs. H + AEC-CM, Figure 5D).

The observed effect of the hydrogel alone can be attributed to its composition, particularly its hyaluronic acid content. HA is well-documented for its immunosuppressive properties and its ability to induce the M2 phenotype in macrophages.^67−69^ This dual functionality of the hydrogel, acting both as a delivery system for the AEC secretome and as an immunomodulatory agent, enhances the therapeutic potential of the combined treatment.^70^ The hydrogel not only facilitates the sustained release of the secretome, but also independently contributes to the immunomodulatory milieu.^68^ This positive effect synergizes with the already validated effect of the secretome alone, as previously observed in the PBMC activation assay.^50,62^ Therefore, the hydrogel acts as more than just a passive carrier; it actively participates in the therapeutic process, providing dual functionality. The combined approach of using the hydrogel-encapsulated secretome showed a clear advantage in promoting anti-inflammatory macrophage polarization, further underscoring its potential in therapeutic applications.

## Conclusion

In this study, an *in situ* gellable iEDDA-cross-linked HA and PEG-based formulation encapsulating AEC-CM was developed. HA-NB and 4-arm PEG-TZ, as well as heparin-modified 4-arm PEG-TZ, precursor polymers were successfully functionalized, producing a hydrogel formulation within minutes after mixing both precursor solutions. The physicochemical characterization confirmed the covalent cross-linking of the formulation, and morphological studies proved that the formulation owned a highly porous inner matrix. Moreover, rheological and swelling studies verified that the fast-cross-linking kinetics, mechanical properties, and low swelling degree qualities of the developed formulation correlated with those observed in other covalently cross-linked hydrogels systems. Heparin-modified hydrogels showed a reduced initial burst release of the protein fraction and different predominant growth factors from AEC-CM, followed by a prolonged sustained release over time in comparison with nonfunctionalized hydrogels.

The enhanced therapeutic efficacy was evident in the significant suppression of PBMC activation, where the combination of the hydrogel with the AEC secretome improved the immunosuppressive effects observed with the secretome alone. Additionally, a pronounced shift in macrophage phenotype toward the M2 state was observed, with the combination inducing a stronger effect compared to the secretome alone. In conclusion, the iEDDA-cross-linked hydrogel formulation not only effectively encapsulates and releases AEC-CM but also amplifies its biological effects, offering a synergistic and potent therapeutic approach for immunomodulation and tissue regeneration.

Overall, this innovative system holds significant potential for advancing regenerative medicine applications.

## Supporting Information Available

H NMR spectra of HA-NB and 4-arm PEG-TZ, calibration curves corresponding to the photoactivated thiol–ene reaction for the determination of functionalization degree of HA-NB, and phenotypic characterization of AEC, as well as the levels of total protein, AREG and VEGF before and after the secretome lyophilization, with its immunomodulatory effect (PDF)
