# Supplementary material for: Development of Amniotic Epithelial Stem Cells Secretome-Loaded In Situ Inverse Electron Demand Diels–Alder-Cross-Linked Hydrogel as a Potential Immunomodulatory Therapeutical Tool
Source: ACS Appl Mater Interfaces. 2025 Jan 2;17(2):2977–90. doi: 10.1021/acsami.4c16659 (PMC11744511; doi:10.1021/acsami.4c16659)
Supplement: Supplementary file 1 — am4c16659_si_001.pdf [file am4c16659_si_001.pdf]

Supporting information

Development of amniotic epithelial stem cells secretome-loaded *in situ* inverse electron demand Diels-Alder-crosslinked hydrogel as a potential immunomodulatory therapeutical tool

Rubén Pareja Tello[1,§,], Adrián Cerveró-Varona[2,§*], Giuseppe Prencipe[2], Giuseppina Molinaro[1], Veronica Pinnarò[3], Arlette Alina Haidar-Montes[2], Alexandra Correia[1], Sami Hietala[4], Johannes Stöckl[3], Jouni Hirvonen[1], Goncalo Barreto[5,6,7], Valentina Russo[2], Barbara Barboni[2,*], Hélder A. Santos[1,8,*]*

[1] Drug Research Program, Division of Pharmaceutical Chemistry and Technology, University of Helsinki, 00014 Helsinki, Finland

[2] Unit of Basic and Applied Sciences, Department of Biosciences and Agro-Food and Environmental Technologies, University of Teramo, 64100 Teramo, Italy

[3] Center for Pathophysiology, Infectiology and Immunology, Institute of Immunology, Medical University of Vienna, 1090 Vienna, Austria

[4] Department of Chemistry, University of Helsinki, 00014 Helsinki, Finland

[5] Clinicum, Faculty of Medicine, University of Helsinki and Helsinki University Hospital, 00014 Helsinki, Finland

[6] Medical Ultrasonics Laboratory (MEDUSA), Department of Neuroscience and Biomedical Engineering, Aalto University, 02150 Espoo, Finland

[7] Orton Orthopedic Hospital, Tenholantie 10, 00280 Helsinki, Finland

[8] Department of Biomaterials and Biomedical Technology, The Personalized Medicine Research Institute (PRECISION), University Medical Center Groningen, University of Groningen, Ant. Deusinglaan 1, 9713 AV Groningen, The Netherlands

[*] Corresponding author: parejatello@helsinki.fi; acerverovarona@unite.it; bbarboni@unite.it; h.a.santos@umcg.nl

[§] These authors contributed equally to the paper

Results and discussion

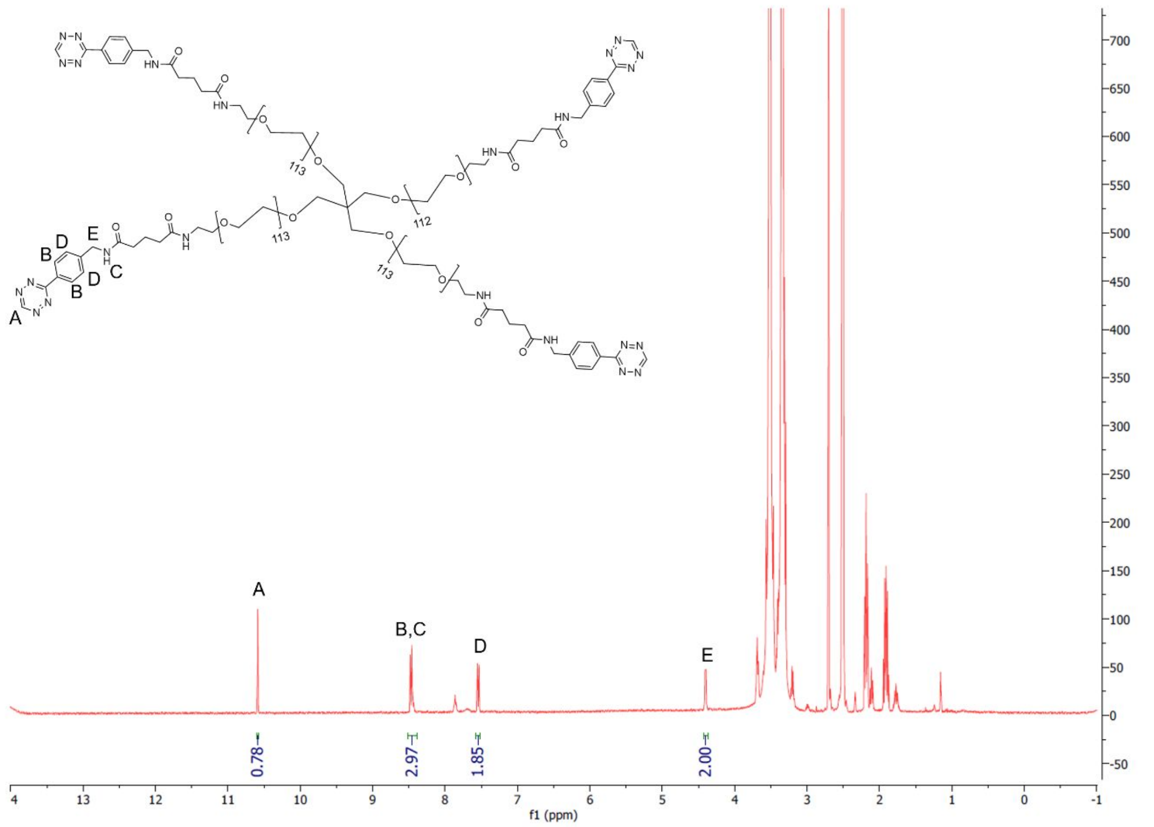

Figure S1. ^{1}H-NMR spectra of 4-arm PEG-TZ.

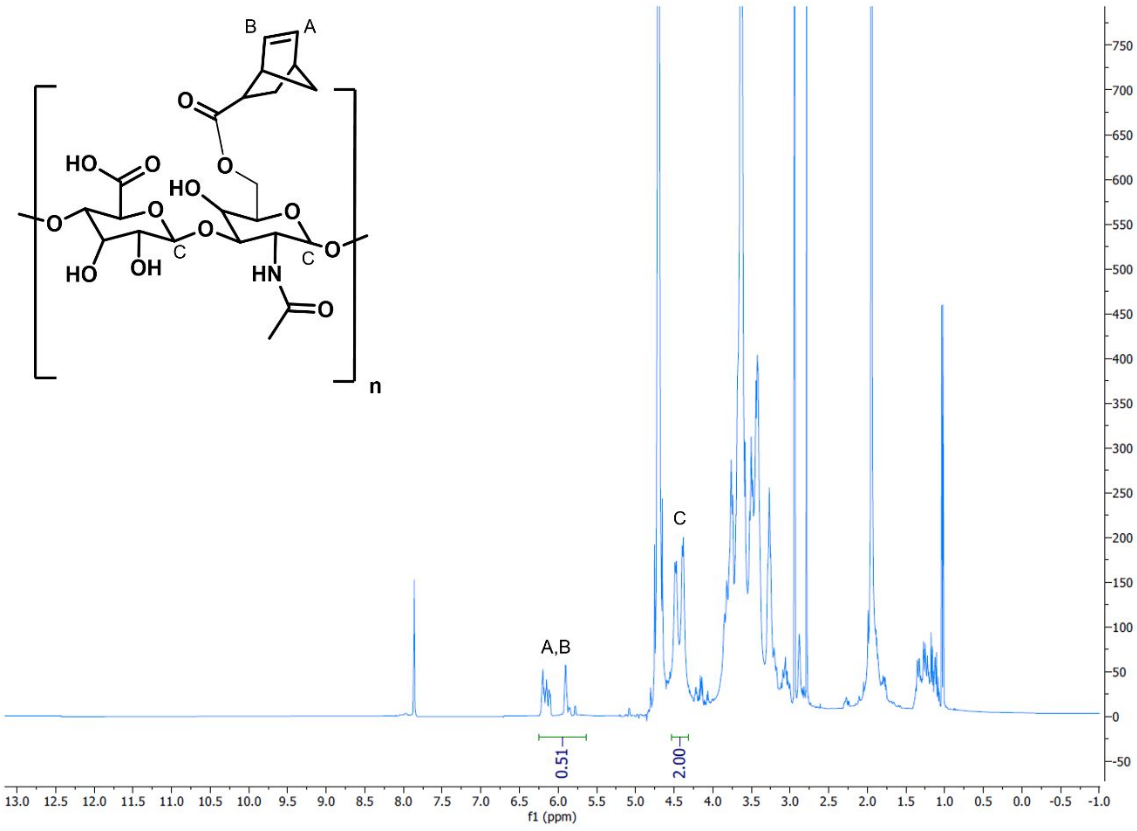

Figure S2. ^{1}H-NMR spectra of HA-NB.

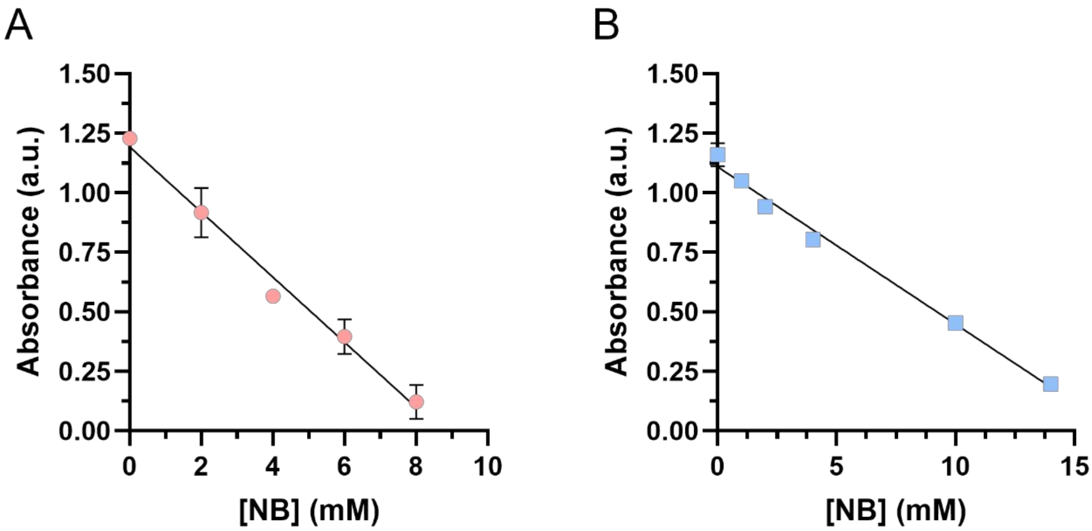

Figure S3. Determination of the functionalization degree of HA-NB via thiol-norbornene photoreaction. (A) Standard curve of carbic anhydride concentration versus the absorbance at 412 nm after reacting with Ellman’s reagent. (B) Curve of HA-NB concentration versus the absorbance at 412 nm after reacting with Ellman’s reagent.

A

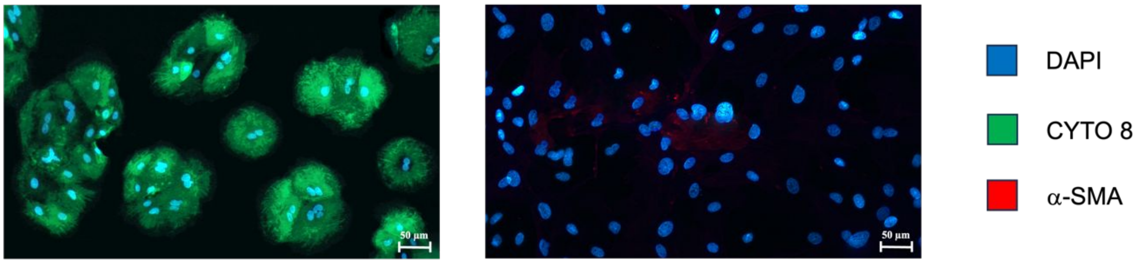

B

	Secretome pre-lyophilization	Secretome post-lyophilization
Total protein (ug/ml)	14.4 ± 1.2	13.8 ± 2.2
AREG (pg/ml)	77.3 ± 8.9	73.5 ± 3.6
VEGF (pg/ml)	45 ± 1.9	48.5 ± 7.9

C

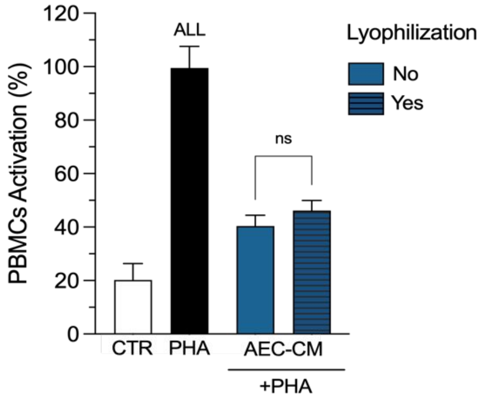

Figure S4. (A) Immunohistology characterization of AEC for the epithelial marker Cytokeratin 8 (CYTO 8) in green, and the mesenchymal marker Alpha Smooth Muscle Actin (αSMA) in red. Nucleus with DAPI in blue. (B) Concentration levels of total protein, AREG and VEGF in the AEC-derived secretome before and after lyophilization. (C) PBMCs activation assay with the AEC-derived secretome before and after lyophilization.
